# Quantitative image analysis of intra-tumoral bFGF level as a molecular marker of paclitaxel resistance

**DOI:** 10.1186/1479-5876-6-4

**Published:** 2008-01-18

**Authors:** Colin T Walsh, Yong Wei, M Guillaume Wientjes, Jessie LS Au

**Affiliations:** 1 College of Pharmacy, The Ohio State University, 500 West 12th Avenue, Columbus, OH, 43210, USA; 2 James Cancer Hospital and Solove Research Institute, The Ohio State University, 300 West 10th Avenue, Columbus, OH, 43210, USA

## Abstract

**Background:**

The role of basic fibroblast growth factor (bFGF) in chemoresistance is controversial; some studies showed a relationship between higher bFGF level and chemoresistance while other studies showed the opposite finding. The goal of the present study was to quantify bFGF levels in archived tumor tissues, and to determine its relationship with chemosensitivity.

**Methods:**

We established an image analysis-based method to quantify and convert the immunostaining intensity of intra-tumor bFGF to concentrations; this was accomplished by generating standard curves using human xenograft tumors as the renewable tissue source for simultaneous image analysis and ELISA. The relationships between bFGF concentrations and tumor chemosensitivity of patient tumors (n = 87) to paclitaxel were evaluated using linear regression analysis.

**Results:**

The image analysis results were compared to our previous results obtained using a conventional, semi-quantitative visual scoring method. While both analyses indicated an inverse relationship between bFGF level and tumor sensitivity to paclitaxel, the image analysis method, by providing bFGF levels in individual tumors and therefore more data points (87 numerical values as opposed to four groups of staining intensities), further enabled the quantitative analysis of the relationship in subgroups of tumors with different pathobiological properties. The results show significant correlation between bFGF level and tumor sensitivity to the antiproliferation effect, but not the apoptotic effect, of paclitaxel. We further found stronger correlations of bFGF level and paclitaxel sensitivity in four tumor subgroups (high stage, positive p53 staining, negative aFGF staining, containing higher-than-median bFGF level), compared to all other groups. These findings suggest that the relationship between intra-tumoral bFGF level and paclitaxel sensitivity was context-dependent, which may explain the previous contradictory findings on the merit of using plasma or urine bFGF level as a prognostic indicator.

**Conclusion:**

The present study established a quantitative image analysis method that enabled the measurement of intratumoral bFGF level in archived tissues. The ability to quantify a potential biomarker provided the opportunity to study the relationship between the biomarker and chemosensitivity in tumor subgroups and thereby enabled hypothesis generation for additional translational research.

## Background

A conventional paradigm in cancer drug development is to identify worthy molecular targets and the corresponding intervening agents in preclinical models, followed by clinical evaluations in human patients. With a few exceptions, the clinical development was done without the knowledge whether the intended targets were present or important for the patients enrolled in clinical trials. This generalist approach has not been highly productive. For example, from 1996 through 2002, 209 anticancer drugs or treatments aiming at 18 newly identified molecular targets (e.g., growth factors, angiogenesis, DNA structure modifications, extracellular matrix proteins, apoptosis-regulatory proteins) entered clinical evaluation, and only 12 drugs/treatments or less than 6% produced survival benefits [1].

An emerging paradigm of matching molecular targeted therapy with patients or diseases with the intended targets has yielded some successes. The most astounding example is imatinib, which has shown significant activity in chronic myelogenous leukemia and gastrointestinal stromal tumor, the two diseases that have the two intended targets of imatinib (Bcr-Abl tyrosine kinase and c-Kit tyrosine kinase) as the respective key lesions [2,3]. On the other hand, most human cancers have multiple lesions in multiple signaling pathways and would be less likely to respond to a single agent targeting a single aspect in the faulty pathways. A more likely scenario is where the intended molecular target can be readily identified and used to preselect patients for evaluation. An example of success in this area is trastuzumab, a humanized monoclonal antibody that binds to the HER2/neu (erbB2) receptor and thereby prevents signal transduction. In HER2-positive metastatic breast cancer patients, addition of trastuzumab to chemotherapy significantly improved the time to progression, response rate, and overall survival [4]. Conversely, while gefitinib, an inhibitor of epidermal growth factor receptor (EGFR) typrosine kianse, improved the objective response rate in non-small cell lung cancer patients, it did not produce survival benefits. A subsequent study identified several qualitative (EGFR mutation status) and quantitative markers (number of EGFR gene copies, EGFR protein level) as potentially important prognostic indicators for response rate and survival [5]. Taken together, these examples illustrate that successful translation of molecular discoveries to useful clinical interventions is possible. The gefitinib example further highlights the potential importance of quantifying the levels of molecular markers.

Our laboratory is interested in evaluating fibroblast growth factors (FGF) as potential targets for overcoming chemoresistance. This is based on our finding that extracellular basic FGF (bFGF) induces broad spectrum chemoresistance in cultured rodent and human prostate cancer cells [6]. This finding is consistent with the findings in small cell lung cancer cells, bladder cancer cells, chronic lymphocytic leukemic cells and fibroblasts, where bFGF causes resistance to multiple chemotherapeutic drugs including etoposide, cisplatin, fludarabine, doxorubicin, methotrexate, hydroxyurea, 5-fluorouracil, paclitaxel, N-(phosphonacetyl)-L-aspartic acid [7-9]. On the other hand, bFGF has also shown the opposite effect and sensitizes breast, prostate, ovarian and pancreatic cancer cells to different chemotherapeutic agents including cisplatin, etoposide, 5-fluorouracil, doxorubicin, carboplatin, and docetaxel [7]. In cancer patients, the role of bFGF expression in clinical prognosis is controversial. In pancreatic cancer, there is no relationship between the intra-tumoral level of bFGF and postoperative recurrence and survival, but an increased FGF receptor expression is associated with shorter survival [10]. A similar observation was made in non-small cell lung cancer patients [11]. There are also reports indicating an opposite relationship in patients for several cancer types. One study showed an association between increased bFGF expression in tumors and shorter survival in node-negative breast cancer patients [12], whereas other studies showed an association between increased intra-tumoral bFGF expression and better prognosis in breast cancer patients [13-17]. Similar (contradictory) results between bFGF expression and prognosis have also been reported for ovarian cancer [18] and pediatric high-grade gliomas [19]. The reasons for the apparently contradicting relationships between bFGF level and prognosis are not clear.

bFGF is a pleotropic growth factor and its levels is affected by multiple processes (e.g., during would healing, angiogenesis). Previous studies on the relationship between bFGF level and chemosensitivity typically test bFGF levels in plasma or urine. Analysis of bFGF level in tumors is theoretically more likely to correlate with the tumor chemosensitivity, but is limited by the lack of accessibility to patient tumor samples. Hence, a method that allows the use of archived tissues may have greater utility, especially if the evaluation is performed retrospectively for, e.g., the purpose of preliminary testing whether a laboratory-generated hypothesis may have clinical utility. A typical method for studying archived tissues is immunohistochemical staining the protein-of-interest and scoring the staining intensity by visual examination. We applied this method to study the relationship, and established an inverse correlation, between intra-tumoral bFGF level and tumor sensitivity to paclitaxel in 96 patient tumors [20]. Because the visual examination method is highly dependent on investigator-defined parameters, there is the potential for unintended bias. Furthermore, the scoring method primarily describes the outcome in discontinuous terms (i.e., yes vs no, more vs less), and therefore has limited utility in situations that require the numerical value of the biomarker.

The present report described the development of a quantitative image analysis-based method to analyze bFGF levels in archived patient tumor tissues. The image analysis results indicated an inverse relationship between intra-tumoral bFGF levels and paclitaxel sensitivity, and enabled the identification of four pathobiological parameters (stage, p53 and aFGF status, higher-than-median bFGF level) that affected the relationship between bFGF levels and paclitaxel sensitivity.

## Methods

### Chemicals and supplies

Bicinchoninic acid and bFGF monoclonal antibody were purchased from Sigma Chemical Co. (St. Louis, MO), Streptavidin-Biotin detection kit from Dako (Carpiteria, CA), cefotaxime sodium from Hoechst-Roussel (Somerville, NJ), DAB (3,3 diaminobenzidine tetrahydrochloride) substrate kit from BioGenex (San Ramon, CA), and collagen gel from Ethicon (Somerville, NJ). All other cell culture supplies were purchased from GIBCO (Grand Island, NY). All chemicals and reagents were used as received.

### Development of image analysis method

The image analysis-based measurement was accomplished in 4 steps: (a) Identified the parameters for image analysis and developed a macro that automated the procedures. (b) Identified the human xenografts tumors that yielded a 100-fold range of bFGF levels as measured by ELISA. (c) Comparison of bFGF levels in xenograft tumors measured by image analysis and ELISA, to obtain standard curves. (d) Using the standard curve to convert the image analysis readings to protein levels.

### Human tumor specimens

Archived tissues of tumors previously studied for paclitaxel sensitivity were used [20]. Of the original 96 patient tumors described in the previous study, 87 (15 bladder, 14 breast, 22 head and neck, 13 ovarian, and 23 prostate) contained sufficient materials for the current study. The pharmacodynamics of paclitaxel effects in these tumors were obtained from previous report [20]. The present study used the control, untreated samples to determine the baseline bFGF level.

### Animal protocols

Establishing standard curves for quantifying the bFGF level required renewable tumor source. This was accomplished by using human xenograft tumors maintained in immunodeficient mice. We screened several human tumor cell lines, i.e., prostate (PC3), pancreatic (MiaPaCa-2 and Hs766T), colon (HT29), ovarian (SKOV3), renal cell carcinoma (RCC), and pharynx (FaDu) (American Type Culture Collection, Rockville, MD). Pilot studies indicated a ~100-fold range in the bFGF levels in these cell lines. The four cell lines that yielded the greatest dynamic range of bFGF levels, i.e., FaDu, HT29, PC3, and MiaPaCa-2, were selected for further studies. Cells were cultured at 37°C in a humidified atmosphere containing 5% CO_2_, in culture medium (PC3 and MiaPaCa-2 in DMEM medium, HT29 in McCoy's 5A plus 0.1% non-essential amino acids, and FaDu in MEM medium plus amino acids) supplemented with 10% fetal bovine serum, 90 mg/ml gentamicin, 2 mM L-glutamine, and 90 mg/ml cefotaxime.

Five-week old mice were purchased from the National Cancer Institute (Bethesda, MD), housed and cared for in accordance with institutional guidelines. Tumors cells were harvested from sub-confluent cultures using trypsin, suspended in serum free medium, and implanted subcutaneously into the flank on both sides of a mouse (2 × 10^6 ^cells/200 μl per injection site). PC3 cells were implanted in male Balbc/nu.nu mice, HT29 in female athymic nude mice, and MiaPaCa-2 and FaDu in male athymic nude mice. Tumors (5–7 mm in length) were harvested and, after removing the non-tumor tissues, were each divided into two halves. One-half was fixed in 10% formalin and embedded in paraffin for immunohistochemical staining for bFGF and image analysis. The other half was used to analyze for bFGF level using ELISA.

### ELISA analysis of bFGF levels

Xenograft tumors were weighed, minced, and sonicated in a mixture of ice cold RIPA lysis buffer (containing 150 mM NaCl, 50 mM Tris (pH 7.4), 1% NP-40, 1% deoxycholate, 0.1% sodium dodecyl sulfate, 2 mM EDTA, 50 mM NaF; Upstate Biochem., Lake Placid, NY), 0.2% Protease Inhibitor Cocktail Set III (CalBiochem, San Diego, CA), and 1 mM phenylmethylsulfonyl-fluoride. Tumor lysates were centrifuged at 14,000 rpm for 15 min and the supernatant was stored at -70°C and subsequently analyzed for bFGF level using an ELISA kit (Oncogene, Cambridge, MA) according to manufacturer instructions. The antibody was murine monoclonal anti-bFGF antibody conjugated to horseradish peroxidase. Detection limit of the assay was 2.5 pg/ml.

### Quantification of FGF levels in tumor tissues using image analysis

Sections of xenograft tumors and archived patient tumors were stained for bFGF using immunohistochemical methods as previously described, with the exception that we used only one antibody dilution (1:50) [20,21]. The inter-day variations in staining intensity were established using the same standard curve samples stained on different days; the average variations in the intensity in individual samples (5 repeats) were 28.1% and the average variation in the resulted standard curve slope was 18.5%.

Images were captured at a magnification of 400× using a Hamamatsu (Hamamatsu-City, Japan) color chilled 3CCD camera attached to a ZEISS (Thornwood, NY) Axiovert 35 microscope, saved in 8-bit TIF format and analyzed in the HSI format (hue, saturation, intensity). The HSI format was preferred over the standard RGB format due to the better separation of the brown (bFGF staining) and blue (hematoxylin counterstain) colors. A macro was written in Optimas^® ^(version 6.51, Media Cybernetics, Silver Spring, MD) to enable unsupervised, high throughput processing of the images. The macro opened each image in turn, applied a user defined threshold to distinguish the brown from the blue staining, extracted the sum of the optical densities (OD) for each image, and converted the data to the corresponding bFGF level using a standard curve.

We quantified 10 images for each of the standard curve tumor samples, and on average 4 images per patient tumor (determined by the amount of available tissues).

### bFGF standard curve

To correct for day-to-day variations in staining intensity, the four standard curve xenograft tumor samples were sectioned and processed together with patient tumor samples. Tumor sections were photographed and the sums of OD were calculated using the macro. For the xenograft tumor samples, the image analysis results (sum of OD values) were plotted against the ELISA results on bFGF levels in respective tumors, to generate a standard curve.

### Statistical analysis

Coefficients of determination (r^2^) were calculated using linear regression (SAS Inc., Cary NC, USA). Confidence intervals around r^2 ^were estimated using Fisher's Z transformation [22].

## Results

### Development of quantitative image analysis method

**Figure 1:**
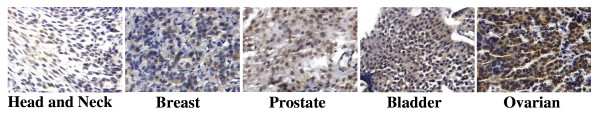
**bFGF staining in patient tumors**. Surgical tumor specimens from patients (n = 87, untreated) were stained for bFGF as described in Materials and Methods. Representative samples of each tumor type are shown. 400× magnification.

Figure 1 shows the bFGF immunostaining in patient tumors. Consistent with our previous finding [20], the location of the staining varied substantially within individual tumors and between tumors. For example, some tumors showed staining exclusively in the cytoplasm or exclusively in the nucleus, whereas some tumors showed staining in both areas. Substantial variations in the staining intensity within an individual tumor, as would be expected due to the heterogeneity in tissues, were also observed. We analyzed images with varying staining characteristics (e.g., strong and weak staining in cytoplasm and nucleus, strong and weak staining in stromal tissues) to identify the dynamic ranges of the HSI parameters (hue, saturation, and intensity). The threshold values for the hue and saturation parameters were set to distinguish the blue stain (for nuclei) from the brown stain (for bFGF). The threshold value for the intensity parameter was set by incrementally changing the range such that the final value found in 20 randomly selected slides (4 from each tumor type) accurately identified the visually bFGF-stained areas while excluding the visually non-stained areas (e.g., cellular debris, blood cells). The selected threshold values were used for subsequent studies.

**Figure 2:**
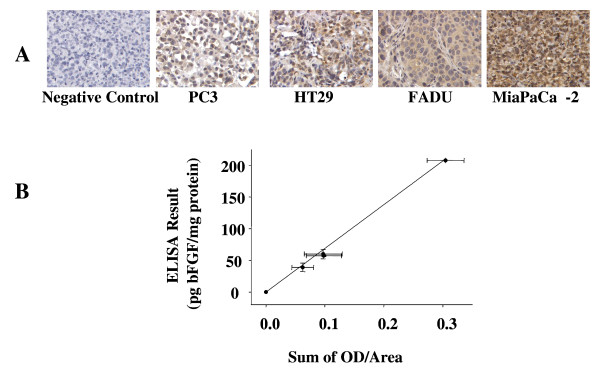
**bFGF standard curve**. (A) Staining of bFGF (brown color) in PC3, HT29, FaDu, and MiaPaCa-2 tumors. Counter-stained with hematoxylin (blue). 400× magnification. The corresponding respective bFGF levels measured using ELISA were 39.0, 57.3, 60.0, and 208 pg/mg total protein. Negative control: MiaPaCa-2 tumor stained with nonspecific, IgG antibody. (B) bFGF standard curve (y = 690.5x; r^2 ^= 0.996, p < 0.001). Mean ± Standard deviations.

We evaluated several quantitative parameters. The use of gray values (mean value, log grey vale) was ruled out because the results obtained using this parameter showed a very narrow range of values and did not correlate with the ELISA results, e.g., tumors (PC3 and MiaPaCa-2) that showed a 4-fold difference in bFGF concentration by the ELISA assay yielded respective gray values of 135 and 129 or a 5% difference. The total area stained for bFGF (positive vs negative) showed a good correlation with the ELISA results (r^2 ^= 0.988, p < 0.005). The Average OD value provided a slightly better correlation (r^2 ^= 0.990, p < 0.005), but did not provide a value for the non-stained area. In comparison, the sum of OD captured the differences in staining intensity and the sum of the positively-stained areas. For example, HT29 and FaDu tumors showed very different bFGF staining patterns, i.e., punctuated, high intensity staining in HT29 vs diffused and less intense staining in FaDu (Figure 2A). But these tumors showed similar sums of OD, in agreement with the similar total bFGF levels measured by ELISA. Furthermore, the sums of OD were linearly correlated with the bFGF levels measured by ELISA, in 4 tumor types (Figure 2B; y = 690.5x; r^2 ^= 0.996, p < 0.001). Subsequent analysis used the sum of OD parameter.

Figure 2B shows a representative standard curve, covering the range of 0 to 208 pg bFGF/mg protein. Three of the four tumors used to obtain the standard curve showed a narrower bFGF concentration range of 0–60 pg/mg protein. The highest level of 208 pg/mg was derived from MiaPaCa-2 tumor. Exclusion of the MiaPaCa-2 tumor did not significantly alter the r^2 ^value. A majority of the patient tumor samples (65/87, 75%) used in the present study showed < 60.0 pg bFGF/mg protein.

### Comparison of visual scoring and imaged analysis results

Table 1 compares the results obtained using the computerized image analysis results to our earlier results established using the conventional, visual scoring method. For bFGF levels in individual tumors, the results from the two methods show a significant, positive correlation (r^2 ^= 0.354, p < 0.0001). For the relationship between bFGF levels and paclitaxel sensitivity in individual tumors, both methods yielded significant correlation, but the image analysis results showed 2–4 times higher r^2 ^values.

**Table 1:** Relationship between bFGF level and paclitaxel activity: Comparison of image analysis and visual scoring results. The linear regression relationships between bFGF levels and the antiproliferation effect of paclitaxel (measured as E_MAX _and IC_30_) were analyzed using SAS. p-values indicate probability associated with r^2^. The 95% confidence interval (95% CI) around r^2 ^is approximated as described previously [22].

**Antiproliferation effect of paclitaxel**	**Relationship between bFGF level and chemosensitivity**		
	
	**r^2^**	**p-value**	**95% CI around r^2^**
**E**_**MAX**_			
Image Analysis	0.208	< 0.0001	0.074–0.370 ^§^
Visual scoring	0.094	< 0.0001	0.011–0.236 ^§^
**IC**_**30**_			
Image Analysis	0.317	< 0.0001	0.160–0.478*
Visual scoring	0.084	< 0.01	0.007–0.222*

^§ ^The r^2 ^for image analysis showed a trend of being higher than the r^2 ^for pathological scoring (p = 0.11).

* The r^2 ^for image analysis (0.317) were outside the 95% CI for pathological scoring (0.007–0.222), indicating a statistically significant improvement in correlation using image analysis (p < 0.05).

### Relationship between bFGF level and paclitaxel sensitivity in individual patient tumors

**Figure 3:**
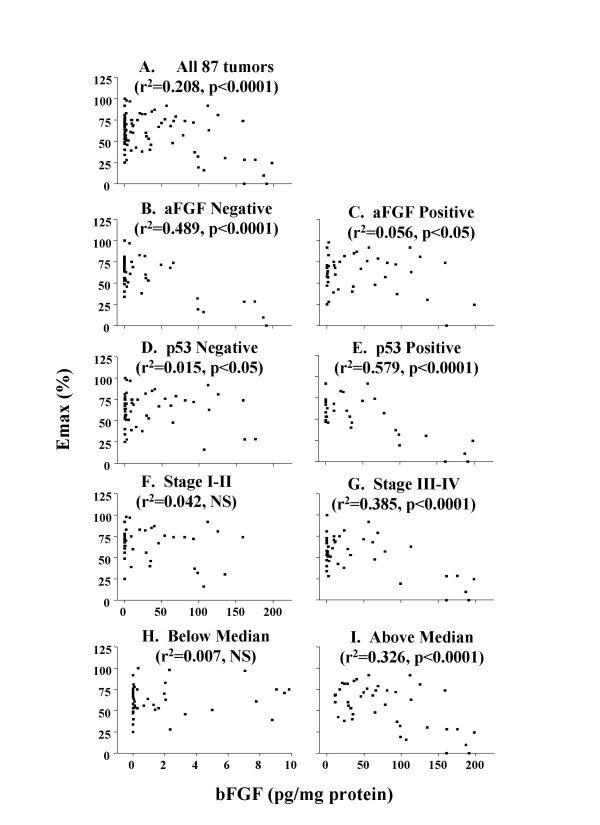
**bFGF levels vs. paclitaxel effects (E_MAX_) in tumor subgroups**. (A) Complete data set (n = 87), (B) tumors with negative aFGF staining (n = 44), (C) tumors with positive aFGF staining (n = 43), (D) tumors with negative p53 staining (n = 54), (E) tumors with positive p53 staining (n = 33), (F) low stage tumors (stage I or II, n = 39), (G) high stage tumors (stage III or IV, n = 48), (H) tumors with bFGF levels less than or equal to the median value of 9.8 pg/mg protein (n = 44), and (I) tumors with bFGF levels greater than the median value (n = 43). Note the different x-axis in panel H (ranged from 0 to 10 pg/mg protein) compared to all other panels (ranged from 0 to 200 pg/mg protein). The r^2 ^and p values for the correlations within each group are provided. The differences between the r^2 ^values between each pair of subgroups (e.g., B vs C, D vs E, F vs G, H vs I) were significant (p < 0.05). The differences between the r^2 ^values between the four subgroups with the high r^2 ^values (i.e., negative aFGF, high stage, positive p53 staining, higher-than-median bFGF levels) and all 87 tumors were also significant (p < 0.05).

**Figure 4:**
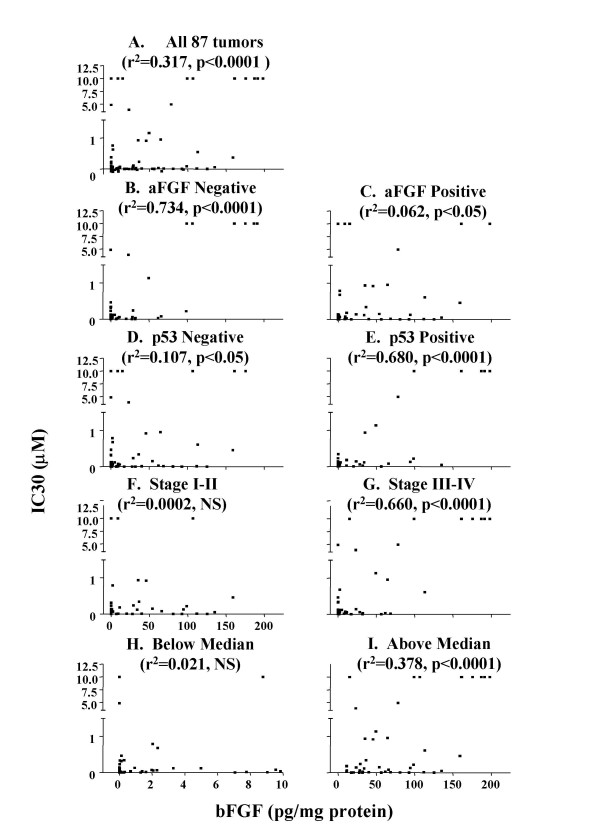
**bFGF levels vs. paclitaxel effects (IC_30_) in tumor subgroups**. (A) complete data set (n = 87), (B) tumors with negative aFGF staining (n = 44), (C) tumors with positive aFGF staining (n = 43), (D) tumors with negative p53 staining (n = 54), (E) tumors with positive p53 staining (n = 33), (F) low stage tumors (stage I or II, n = 39), (G) high stage tumors (stage III or IV, n = 48), (H) tumors with bFGF levels less than or equal to the median value of 9.8 pg/mg (n = 44), and (I) tumors with bFGF levels greater than the median value (n = 43). Note the different x-axis in panel H (ranged from 0 to 10 pg/mg protein) compared to all other panels (ranged from 0 to 200 pg/mg protein). The r^2 ^and p values for the correlations within each group are provided. The differences between the r^2 ^values between each pair of subgroups (e.g., B vs C, D vs E, F vs G, H vs I) were significant (p < 0.05). The differences between the r^2 ^values between the four subgroups with the high r^2 ^values (i.e., negative aFGF, high stage, positive p53 staining, higher-than-median bFGF levels) and all 87 tumors were also significant (p < 0.05).

Data on tumor sensitivity to the antiproliferation and apoptotic effects of paclitaxel were obtained from our previous report [20]. The antiproliferative effects were quantified in terms of the drug concentration required to produce 30% inhibition of bromodeoxyuridine labeling index (IC_30_) and the maximum inhibition of the same parameter (E_MAX_). bFGF levels in tumors were significantly and inversely correlated with tumor sensitivity to the antiproliferation effect of paclitaxel (Figures 3 and 4). No relationship was observed between bFGF levels and maximum apoptotic index (data not shown).

### Effects of tumor pathobiological parameters on the relationship between bFGF level and paclitaxel sensitivity

The tumor pathobiological data of patient tumors were obtained from our previous report [20]. Analysis of the relationship between bFGF level and tumor sensitivity to the antiproliferation effect in tumor subgroups revealed significantly better correlations (i.e., higher r^2 ^values) in stage III-IV, p53-positive and aFGF-negative tumors, and tumors with higher-than-median bFGF level (Figures 3 and 4), compared to low stage (I-II), p53-negative, aFGF-positive, and low bFGF tumors. The median bFGF level was 9.8 pg/mg protein. No differences were observed for the other pathobiological parameters (i.e., high or low grade, high or low levels of p-glycoprotein or Bcl-2).

## Discussion

Biomarkers are gaining importance in the development and usage of molecular medicines. The present study presents a computerized image analysis method for quantifying protein biomarkers in archived, paraffin-embedded clinical samples. The two advantages of using immunohistochemical staining for protein-of-interest are the availability of archived samples and, because the 3-dimensional structure is maintained, the spatial or subcellular distribution information that is not available in methods that use tissue homogenates or extracts (e.g., ELISA, Western blotting).

Our image analysis results indicate statistically significant correlation between bFGF level and the antiproliferation effect of paclitaxel (measured as E_MAX _and IC_30_), but no relationship between bFGF level and the maximum apoptotic index. Both findings are consistent with our earlier findings using the conventional, visual scoring method. However, while both methods yielded the same finding of an inverse correlation between bFGF level and tumor sensitivity to the antiproliferation effect of paclitaxel, the image analysis method provided more robust data and presented several additional advantages, as follows.

The visual scoring method was semi-quantitative and based on the differential staining intensity at three different antibody concentrations. For example, a tumor showing no staining at the highest antibody concentration was scored as negative, a tumor showing positive staining at different antibody concentrations were scored as one, two or three pluses. In comparison, the current method required using a single antibody dilution, and, because the quantitative image analysis results were based on the results obtained from simultaneously stained standard curve samples, was not subjected to or affected by inter-day variability in staining intensity. Furthermore, the image analysis method applied the same analysis parameters to all samples and therefore minimized the potential operator bias.

The image analysis method, by measuring bFGF levels in individual tumors, provided values for each of the 87 tumors. In comparison, the visual examination results provided readings of relative intensity which provided only 4 scores (negative and three scores of positive staining). This, in turn, enabled the analysis of the relationship between bFGF level and tumor sensitivity to the antiproliferation effect of paclitaxel in subgroups of tumors with different pathobiological properties; the results show better correlations in four pathobiological subgroups (late stages, positive p53 staining, negative aFGF staining, higher-than-median bFGF level), compared to all other groups. These findings, in turn, improved our ability to extract information and enabled the generation of the following hypotheses. As p53 immunostaining is generally accepted as indicative of mutated p53 [23] and high stage tumors are generally more aggressive compared to low stage tumors, we propose that the effects of bFGF on paclitaxel resistance are more pronounced in the more aggressive tumors with p53 mutation, or, alternatively, the bFGF effects are masked by the presence of functional, wild type p53. The finding of the apparently better correlation of bFGF level and chemosensitivity in aFGF-negative tumors was unexpected in view of our earlier finding that aFGF amplifies the chemoresistance conferred by extracellular bFGF, in cultured monolayer cells [24]. Multiple possible reasons can explain this difference, including the microenvironment and heterogeneity in human tumors compared to cultured cells. Based on the better correlation in the high bFGF-expressing tumors, we speculate that there is a threshold bFGF level above which bFGF has a role in chemoresistance.

Our finding of a lack of correlation between bFGF level and the apoptotic effect of paclitaxel in patient tumors suggests that bFGF level may not predict for tumor shrinkage, a conventional method of measuring patient response to chemotherapy. Finally, the current finding that the relationship between intra-tumoral bFGF levels and tumor sensitivity to the antiproliferation effect of paclitaxel was dependent on the status of multiple tumor pathobiological parameters suggests that the previous contradictory findings on the merit of using plasma or urine bFGF level as a prognostic indicator may be partly due to heterogeneity in patient tumors.

## Conclusion

The present study established a quantitative image analysis method that enabled the measurement of intra-tumoral bFGF level in archived tumor samples. The ability to quantify a potential biomarker provided the opportunity to study the relationship between the biomarker and chemosensitivity in subgroups with different tumor pathobiological parameters and thereby enabled further hypothesis generation, a process that may have implications for translational research.

## List of Abbreviations Used

aFGF: acidic fibroblast growth factor; bFGF: basic fibroblast growth factor; E_MAX_: maximal inhibition of bromodeoxyuridine labeling; EGFR, epidermal growth factor receptor; IC_30_: drug concentration needed to produce 30% inhibition of bromodeoxyuridine labeling; OD: optical density; r^2^, coefficient of determination.

## Competing interests

CTW and YW declare that they have no competing interests. JLSA and MGW are applying for patents to using bFGF as a biomarker for chemotherapy (since 2000).

## Authors' contributions

CTW designed and implemented the study including execution of all experiments involving generation of standard curves and image analysis, performed the statistical analysis and interpreted the correlation data, drafted the manuscript.

YW contributed to the statistical analysis and interpretation of the data and the correlations and helped draft the manuscript.

GW contributed to the statistical analysis and helped draft the manuscript.

JLSA conceived the study, oversaw and participated in the study design and coordination, and drafted the manuscript.

All authors read and approved the final manuscript.
